# The Fatigue Behaviors of a Medium-Carbon Pearlitic Wheel-Steel with Elongated Sulfides in High-Cycle and Very-High-Cycle Regimes

**DOI:** 10.3390/ma14154318

**Published:** 2021-08-02

**Authors:** Lu Liu, Yifan Ma, Shisen Liu, Shengnan Wang

**Affiliations:** 1 Department of Aeronautical Structural Engineering, School of Aeronautics, Northwestern Polytechnical University, Xi’an 710072, China; yifan.ma@mail.nwpu.edu.cn (Y.M.); wangshna@nwpu.edu.cn (S.W.); 2 Department of Aircraft Design, School of Aircraft, Xi’an Aeronautical University, Xi’an 710000, China; 3 State Key Laboratory of Gas Disaster Monitoring and Emergency Technology, China Coal Technology and Engineering Group Chongqing Research Institute, Chongqing 400037, China; tian8935045@126.com

**Keywords:** wheel steel, very-high-cycle fatigue, stress ratio, inclusion, pearlite

## Abstract

The effects of stress ratio (*R*), loading condition, and MnS inclusion on the fatigue behavior of a medium-carbon pearlitic wheel-steel were investigated by a combination of rotating (frequency of 52.5 Hz, 10^3^–10^8^) bending and ultrasonic (frequency of 20 kHz, 5 × 10^4^–10^9^) axial cycling tests in high-cycle and very-high-cycle regimes. All the S-N curves present horizontal asymptotic shapes and have clear fatigue limits. The fatigue limits (260–270 MPa) for *R* = −1 obtained by ultrasonic test are almost 140–150 MPa lower than that (400–410 MPa) obtained by rotating bending, and the limit values of *R* = 0.3 are almost in the range of 195–205 MPa. For rotating bending, the fatigue fractures were originated from the surface matrix of the specimen. Whereas for ultrasonic fatigue, both surface and interior crack initiation occurred, and cracks were all initiated from MnS inclusions regardless of stress ratios. The finite element method was employed to study the influence of MnS inclusions on crack initiation and propagation. The results show that high stress concentrates on the sides of the elliptical MnS inclusion rather than the tip of the inclusion.

## 1. Introduction

As a wheel material, medium-carbon pearlitic steel has been extensively applied in high-speed railway designed to have a service life of 2.4 million kilometers. That is, the wheels must experience 5 × 10^8^–10^9^ loading cycles with no failure [1], which belongs very-high-cycle fatigue (VHCF, failure beyond 10^7^ loading cycles). The performance of high-strength steels in the VHCF regime has drawn considerable attention in last tens of years [2,3,4,5,6]. Specifically, the crack initiation site and early growth path for VHCF are different from that of low-cycle fatigue (LCF) and high-cycle fatigue (HCF). In LCF and most HCF cases of steel, fatigue cracks are initiated from the specimen surface; in contrast with VHCF cases, cracks usually originated from nonmetallic inclusions or other inhomogeneities in the specimen interior [7,8]. However, compared with high-strength steels, the studies of pearlitic steels in VHCF regime are still rarely published. Thus, it is necessary to investigate the fatigue behavior of pearlitic wheel-steels to ensure a safe and economic application of high-speed railway in VHCF service condition.

Some experimental facilities have been applied for the VHCF tests, such as rotating bending (RB), ultrasonic loading (UL) and other axial cycling machines (servo-hydraulic and electromagnetic resonance). Since the VHCF testing consumes too much time at low frequency, ultrasonic fatigue testing (20 kHz) devices are often used, but the increase in loading frequency will affect the obtained VHCF propety, for which several investigations have been reported [9,10,11,12,13,14,15,16,17,18]. Guennec et al. [12] conducted fatigue tests at frequencies of 0.2 Hz, 2 Hz, 20 Hz, 140 Hz, and 20 kHz for a structural steel and the result exhibited that the fatigue strength increased with the applied loading frequency. Bayraktar et al. [13] carried out fatigue tests at the frequencies of 30 Hz and 20 kHz for pearlitic steels: Results for D38MSV5S (0.38 wt% C) showed that loading frequency has little influence on VHCF behavior, whereas for XC70 (0.67 wt% C), the fatigue performance under ultrasonic loading is better than that under the conventional frequency. Recently, Li et al. [16] studied HCF and VHCF behaviors at the frequencies of 150 Hz and 20 kHz for medium-carbon pearlitic wheel-steels and the fatigue performance at 150 Hz show a little better than that at 20 kHz. In summary, loading frequency is indeed an influencing factor for the fatigue behavior of metals and alloys; the effect of loading frequency on VHCF properties of pearlitic wheel-steels requires further investigation.

Nonmetallic inclusions have an important influence on the fatigue failure of steels relying on their distribution (size, shape and location), chemical composition and adhesion to the matrix [16,19,20,21,22,23,24]. Generally, fatigue cracks often initiate from oxide inclusions (such as alumina and calcium oxide) rather than sulfide inclusions (such as MnS) because the former are hard and brittle, whereas the latter are ductile and deformable [22,23]. Li et al. [16] studied the effects of nonmetallic inclusions on the fatigue performance of four wheels designed with different sulfur and similar ultra-low oxygen contents. In their research, only oxides were observed on the surface within the crack initiation area, whereas no sulfide was detected. Dhua et al. [22] discovered that: as the value of oxide volume fraction increases from 0.06 to 0.21%, the fatigue limit gradually decreases in a pearlitic rail steel. Although there is no direct correlation between the fatigue limit and the value of sulfide volume fraction (0.15–0.27%), the MnS inclusions with elongated shape were captured at the crack initiation regions in some cases. However, Liu et al. [23,24] performed fatigue tests under four-point loading on a servo-hydraulic testing frame at a loading frequency of 30 Hz for rail steels and found that that fatigue cracking is prone to occur at the edge of the elongated inclusions owing to higher stress concentration than the round inclusions. In the present study, elongated inclusions were also found at crack initiation sites in wheel-steels; its effect on VHCF properties is discussed in depth.

Many researchers [14,15,18,25,26,27,28,29] reported the HCF and VHCF performances of different alloys with smooth specimens at different values of stress ratio (*R*). For example, Sakai et al. [25] surveyed the VHCF behavior of a high carbon chromium bearing steel with axial cyclic loading and discussed the mean stress effect with a bilinear model. Kovacs et al. [26] reported that the continuous S-N curves of low-carbon steel present the same tendency for all the stress ratios. Gao [28] investigated the effects of the notch and stress ratio on the HCF property of high-strength steel. They used two factors to visualize the sensitivity of notch stress concentration and stress ratio. Moreover, Sander et al. [29] investigated the effects of stress ratio and variable stress on the fatigue behavior of medium-carbon steel; the results revealed that the slope of the continuous S-N curve increases with decreasing value of stress ratio, and the fatigue lives are smaller under the loading condition of *R* = −1 than for *R* = 0. However, the effect of *R* value on the HCF and VHCF behaviors of medium-carbon pearlitic steels has rarely been investigated.

In the current work, fatigue testing of medium-carbon pearlitic wheel-steel was performed with rotating bending (52.5 Hz) and ultrasonic axial loading (20 kHz) machines to clarify the effects of loading condition and frequency on HCF and VHCF behaviors. The effect of stress ratios was also examined for two different values, *R* = −1 and *R* = 0.3, via ultrasonic cycling. Scanning Electron Microscopy (SEM) was used to capture fractographic features of the fatigue fractured specimens. Moreover, the effect of elongated MnS inclusions on the fatigue behaviors was also studied by using Energy Dispersive X-ray analysis (EDX).

## 2. Test Material and Experimental Procedure

### 2.1. Test Material

**Figure 1:**
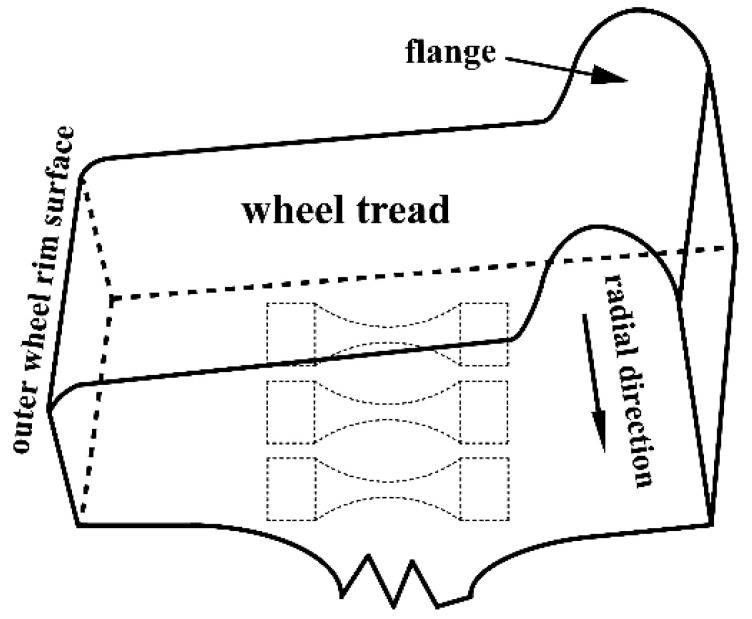
Schematic of wheel rim.

**Figure 2:**
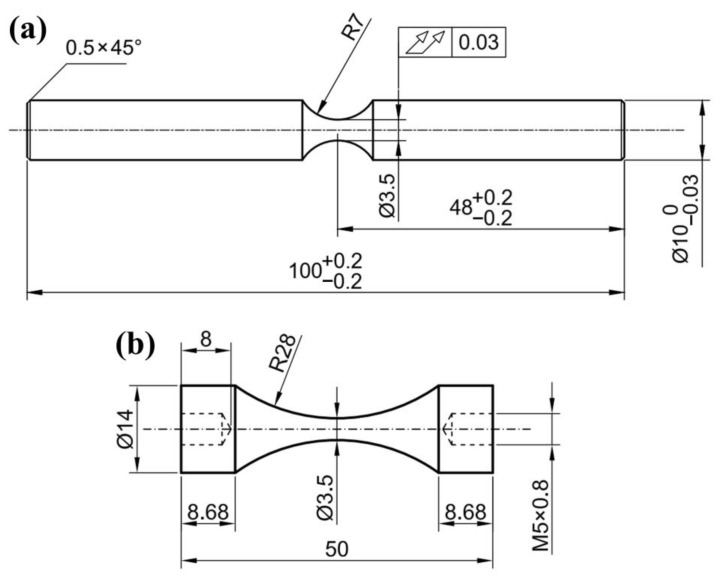
Specimen shapes and dimensions (mm) used in fatigue tests: (**a**) rotating bending; (**b**) ultrasonic axial loading.

The material used in this study is a medium-carbon pearlitic wheel-steel (Maanshan Iron & Steel Company, Maanshan, Anhui, China) with the chemical compositions 0.6 C, 0.27 Si, 0.65 Mn, and 0.036 P in weight percent (Fe balance). These railway wheels were produced with electric arc furnace melting—ladle furnace refining—vacuum degassing, and continuous casting process. After casting, the steel billets were hot forged and hot rolled into wheels, and subsequently, the wheels were austenitized at 850–900 °C for 1 h and quenched (so-called “rim chilling”), followed by tempering at 500 °C for 2 h. The samples for microstructural observation, tensile and fatigue specimens were cut, lifted out and machined in a rolling direction from the rim of the wheels to 15 mm below the tread surface, as shown in Figure 1. The geometries of rotating bending and ultrasonic fatigue testing specimens were shown in Figure 2. All the specimen surfaces were polished with 800 grit sandpaper. Before the fatigue test, the tensile properties of the material were acquired from four cylindrical specimens with a diameter of 10 mm in the gauge length using the servo-hydraulic testing machine by a quasi-static tension test with a cross-head speed of 1 mm/min to measure the stress-strain curve of the tested steels at room temperature and in air.

### 2.2. Experimental Procedure

The fatigue test under conventional frequency was conducted at room temperature and in ambient air by using a rotating bending machine (GIGA QUAD, Yamamoto, Osaka, Japan) with four cantilever axes, which worked at 3150 rpm (52.5 Hz) with the stress ratio of *R* = −1.

Same as the descriptions from Refs. [14,15], an ultrasonic fatigue test was performed via a axial loading machine (GF20-TC, Lasur, Paris, France) with a working frequency of 20 kHz at room temperature and in ambient air, which requires the specimen frequency to be within the range of input incitation frequency (20 k ± 500 Hz) of the piezoelectric ceramic resonator. Before the test, the ultrasonic machine was calibrated by measuring the vibration amplitude at the end of the amplifying horn via a rangefinder with an eddy current displacement sensor (Shimadzu, Kyoto, Japan). The specimen was fully cooled by the cold compressed air during the entire process of an ultrasonic fatigue test. The ultrasonic machine was mounted onto a tensile machine (capacity 20 kN, Tianshui Hongshan, Tianshui, Gansu, China) to realize the loading condition of stress ratio *R* = 0.3. In the case of *R* = −1, the cyclic stress was solely applied by the ultrasonic machine; for that, no mean stress was added.

After the fatigue testing, fracture surfaces of all fatigue failed specimens were properly examined by a field-emission type of SEM (QUANTA 200 FEG, FEI, Hillsboro, OR, USA), and the chemical compositions underneath the fracture surface of crack initiation spots (inclusions or inhomogeneities) were examined by EDX (X-max 150, Oxford Instrument, Abingdon, UK) equipped with SEM. Based on the SEM photos, the sizes of inclusions were measured with ImageJ software (1.53a, 2020, National Institutes of Health, Bethesda, MD, USA). The failure types and crack origination sites were identified on the fracture surfaces.

In addition, two specimens of different stress ratios *R* = −1 and 0.3 that failed in the VHCF regime were selected to characterize the microstructure and to determine the characteristics of fracture surface profile within the crack initiation region. Extracted profile samples from the neighboring area around the inclusions on the fracture surfaces were prepared by a focused ion beam (FIB) technique with an FIB-SEM dual-beam system Helios Nanalab 600i (FEI, Hillsboro, OR, USA). These samples were carefully examined via a Transmission Electron Microscopy (TEM) Tecnai G2 F30 S-Twin (FEI, Hillsboro, OR, USA), in which the detection domain of SAD (Selected electron Area Diffraction) is approximately 200 nm in diameter. Finally, Transmission Kikuchi Diffraction (TKD, NordlysNano, Oxford Instrument, Abingdon, UK) technique was applied for further quantitative analysis of crystal orientation and grain size distribution with a step size of 15 nm.

## 3. Results and Discussion

### 3.1. Microstructural Observations

**Figure 3:**
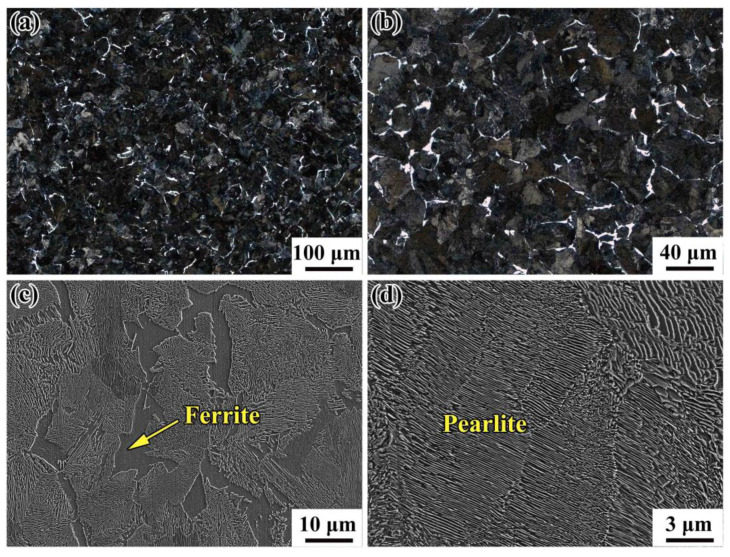
Microstructure of the wheel-steel: (**a**,**b**) OM (optical microscopy) images of pearlite domains (black regions) and proeutectoid ferrite domains (white regions); (**c**,**d**) the SEM (scanning electron microscopy) images.

**Figure 4:**
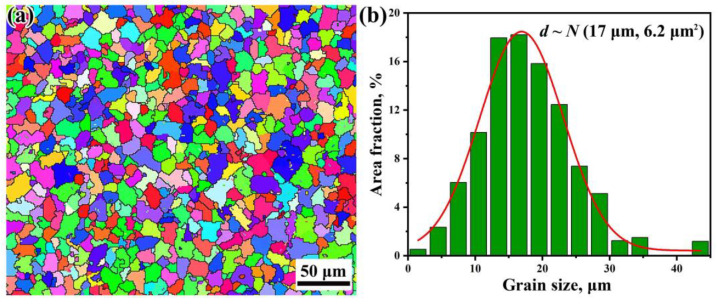
EBSD (electron backscatter diffraction) map of the grains (**a**) and its statistical distribution of the effective grain size (**b**).

Figure 3 shows the typical microstructures of the wheel steels observed via optical microscopy (OM, Axiovert 200 MAT, Zeiss, White Plains, NY, USA) and an SEM, both containing pearlite domains (black regions) and proeutectoid ferrite domains (white regions) which were mainly nucleated along the grain boundary of the prior austenite. For the lamellar structure of pearlite, the interlamellar spacing is approximately 0.13–0.15 μm, which was measured by the linear intercept method from SEM images. The results based on EBSD (Electron Backscatter Diffraction) technique are presented in Figure 4 via a field-emission typed SEM (Merlin, Zeiss, White Plains, NY, USA) equipped with a NordlysNano detector (Oxford Instrument, Abingdon, UK) showing the distributions of crystal orientation and grain size. Since the pearlite colony size can be approximately regarded as independent of the prior austenite, we only measured the pearlitic block sizes. Therefore, the block size were determined as the domain surrounded by high angle (>15°) boundaries of ferrite on EBSD mappings, as shown in Figure 4a. Note that for the medium-carbon pearlitic steel, the proeutectoid ferrite was also counted in the measurement of the block size, and the influence of area fraction was considered [30]. As shown in Figure 4b, the weighted average effective grain size of the wheel steel is calculated to be 17 μm, and the effective grain size *d* satisfied a Normal distribution *N* (17 μm, 6.2 μm^2^).

The yield strength of the medium-carbon pearlitic steel is 590 MPa, and the ultimate tensile strength (UTS) is 910 MPa, the data of which are obtained from four cylindrical specimens with a dog bone shape (gauge length: 50 mm, diameter: 10 mm).

**Figure 5:**
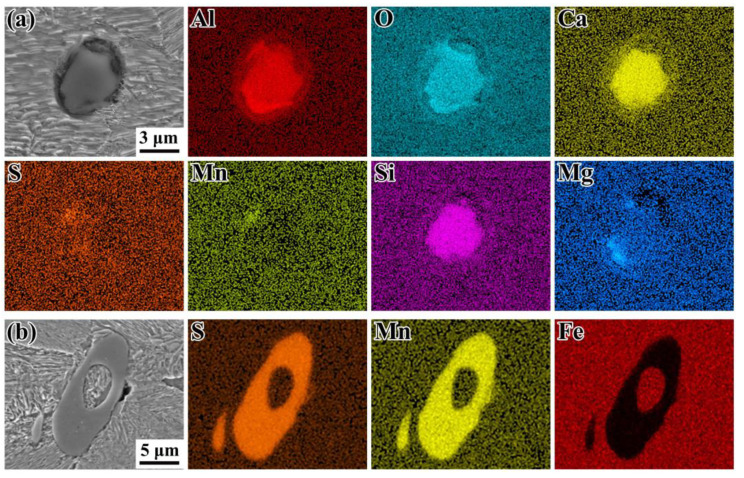
SEM images and EDX (energy dispersive X-ray analysis) maps of the oxysulfides: (**a**) Al_2_O_3_ surrounding oxide, (**b**) elongated MnS inclusion.

Figure 5 shows electron micrographs of representative compositions of the oxide inclusions and sulfide inclusions in the metallographic samples, which were measured by the automated SEM/EDX Aspex analysis. The representative oxide inclusions were mainly Al_2_O_3_, CaO, and SiO_2_ with spherical shapes, as shown in Figure 5a. The representative sulfide inclusions were almost MnS with elongated or ellipsoidal shapes, as shown in Figure 5b.

### 3.2. S-N Data

**Figure 6:**
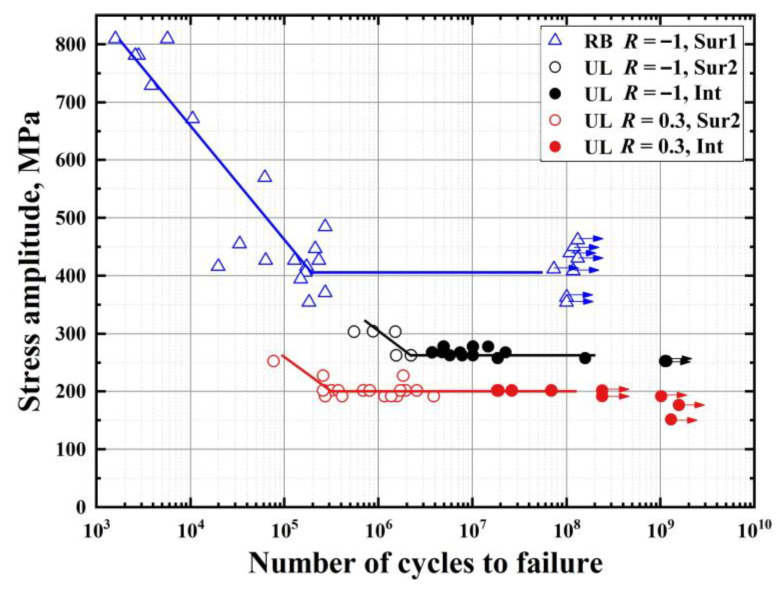
S-N curves of tested specimens under rotating bending and ultrasonic axial cycling with stress ratios of −1 and 0.3. (RB: rotating bending, UL: ultrasonic loading, Sur1: surface crack initiation without inclusion, Sur2: surface crack initiation with inclusion, Int: internal inclusion, symbol with arrow: not broken).

Figure 6 presents the S-N data obtained by rotating bending fatigue tests and ultrasonic fatigue tests in terms of stress amplitude *σ*_a_ versus failure cycles *N*_f_. Stress ratios of *R* = −1 and 0.3 were both measured in the ultrasonic fatigue tests. It can be seen that all S-N curves exhibit a horizontal asymptote and show a clear fatigue limit, which are typical of S-N curves for carbon steel. However, the S-N data for the three groups present very different features. For the rotating bending, all specimens failed between 10^3^ and 10^6^ cycles, covering the LCF and HCF regimes. No specimen failure occurs beyond 10^6^ cycles; this indicates that there are similar values of fatigue limits at 10^7^ cycles and 10^8^ cycles. The values are in the range of 400–410 MPa. For the ultrasonic fatigue tests, the cycles to fatigue failure of all specimens are mainly between 10^5^ cycles and 10^8^ cycles, covering the HCF and VHCF regimes. When compared with the rotating bending results, there is a sharp decrease in fatigue data happening in the ultrasonic fatigue tests. The fatigue limits (260–270 MPa) for *R* = −1 are approximately 140–150 MPa lower than that (400–410 MPa) obtained by rotating bending. For the stress ratio of 0.3, the fatigue strength continues to decline and the fatigue limits are almost in the range of 195–205 MPa.

Ostensibly, the loading frequency (ultrasonic versus conventional) and types (axial cycling versus rotating bending) greatly influence these behaviors. On the one hand, this result is partly due to the heat generation accumulated within the specimen under the ultrasonic frequency in pearlitic steels. The temperature rise is mainly associated with inelastic effects, such as plastic deformation, internal friction [31] and so on. The temperature was not continuously measured during the whole loading process in real-time. However, we measured the temperature at several key moments, such as when the global plastic deformation occurred (the specimen surface turned black), when the load cycles reached a certain number (integer multiples of 10^7^) and when the fatigue fracture occurred. According to Bathias [32], the temperature rise before crack growth under ultrasonic loading is less than 80 °C for carbon steels. The temperature can rise more than 200 °C when the crack growth takes place even if cooling systems are used. The temperature rise accelerates the crack growth, and thus the fatigue limit will decrease. Another reason was attributed to the different maximum size of the nonmetallic inclusions inducing fatigue failure in the different control volume for the different loading conditions. Normally, the interface between a relatively large inclusion and the matrix is more prone to debonding due to higher stress concentration; thus, fatigue crack initiation tends to originate at the inclusions within the specimen interior. Considering that the control volume of a specimen for rotating bending is significantly smaller than that for ultrasonic loading, this led to the maximum size of nonmetallic inclusions under ultrasonic loading being larger than under rotating bending [33]. Lastly, it is emphasized that the stress gradient within the specimen cross-section plays a key role in the rotating bending fatigue.

Figure 6 also contains information on fatigue crack initiation sites. For the rotating bending fatigue tests, surface crack initiation dominates fatigue failures for all specimens before 10^6^ cycles, i.e., LCF and HCF regimes; this is mainly due to the influence of the stress gradient [34,35] and the size effect of smaller control volumes [33,36] in rotating bending. For the ultrasonic fatigue tests, surface crack initiation still govern fatigue failure for all specimens before 5 × 10^6^ cycles, i.e., HCF regimes. By contrast, internal crack initiation takes over the failure type beyond 5 × 10^6^ cycles, i.e., HCF and VHCF regimes. Moreover, the stress ratio of −1 had a larger number of failed specimens with internal crack initiation compared with the stress ratio of 0.3; this indicates that stress ratios influence the fatigue crack initiation location.

### 3.3. SEM Observation and Analysis

**Figure 7:**
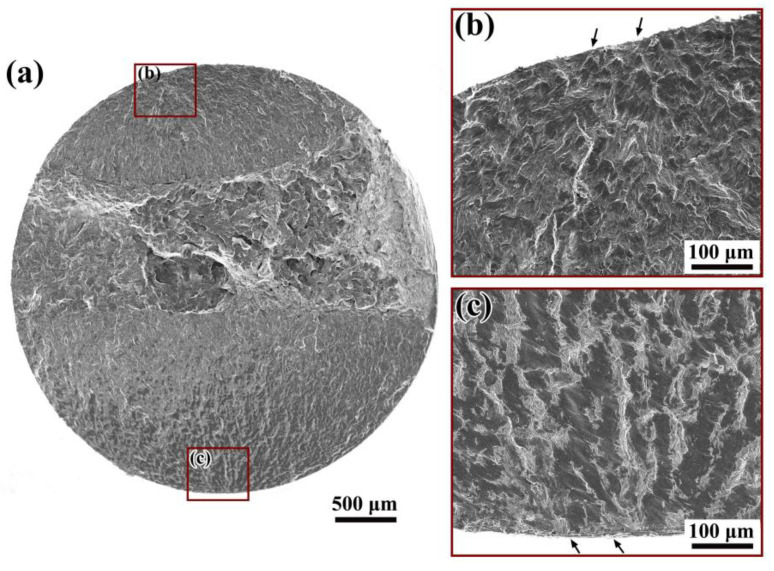
Surface slip-induced crack initiation under rotating bending loading, *σ*_a_ = 415 MPa, *N*_f_ = 1.72 × 10^5^ cycles: (**a**) whole fracture surface, (**b**,**c**) details for crack initiation sites.

The fracture surfaces of all fatigue failed specimens were investigated using SEM. Figure 7 shows a typical fractography of specimens subjected to rotating bending, during which the specimens failed in the HCF regime. Note that all fatigue cracks of the specimens were initiated from the surface microstructure for the rotating bending test. No pre-existing inclusions were observed at the crack initiation sites, and the crack nucleation was probably due to localized deformation at the specimen surface.

**Figure 8:**
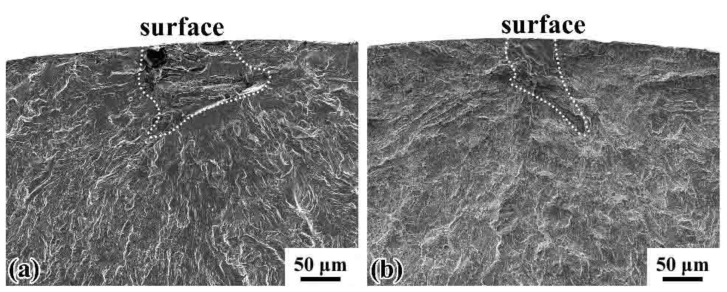
Typical fracture morphologies of surface crack initiation by ultrasonic fatigue test: (**a**) *R* = −1, *σ*_a_ = 263 MPa, *N*_f_ = 1.55 × 10^6^ cycles; (**b**) *R* = 0.3, *σ*_a_ = 227 MPa, *N*_f_ = 1.83 × 10^6^ cycles.

**Figure 9:**
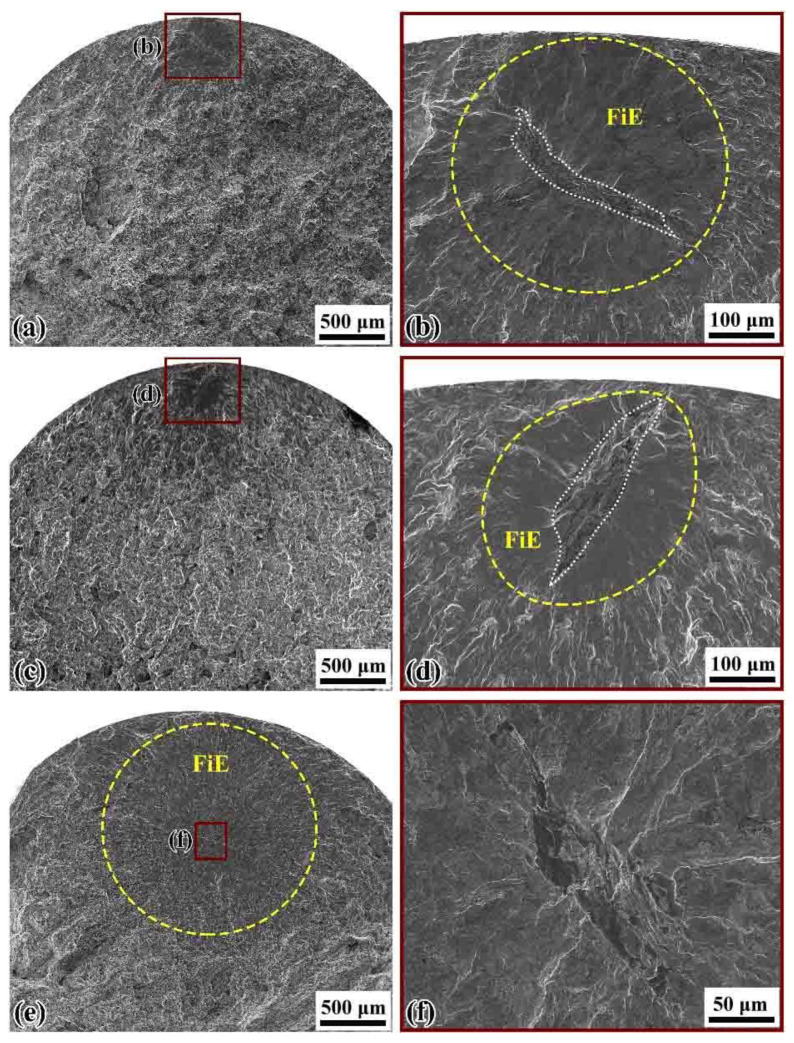
Typical fracture morphologies of internal crack initiation by ultrasonic fatigue test: (**a**) *R* = −1, *σ*_a_ = 262 MPa, *N*_f_ = 7.78 × 10^6^ cycles; (**c**) *R* = −1, *σ*_a_ = 258 MPa, *N*_f_ = 1.01 × 10^7^ cycles; (**e**) *R* = 0.3, *σ*_a_ = 202 MPa, *N*_f_ = 1.84 × 10^7^ cycles; while magnified views of inclusions inducing final failure are shown in (**b**,**d**,**f**), respectively. FiE: “fish-eye” morphology.

For the ultrasonic fatigue tests, both surface and interior crack initiation occurred, and the cracks were all initiated from inclusions regardless of stress ratios, suggesting the nonmetallic inclusions as dominant factor for HCF and VHCF. The fracture surfaces and S-N curves indicate that the fatigue strength and the fatigue crack initiation for the wheel steels are sensitive to nonmetallic inclusions. Figure 8 shows two surface cases of fatigue crack initiation under ultrasonic axial loading at *R* = −1 and 0.3 in HCF regime, respectively. The inclusion at the crack initiation sites was irregular and formed a sort of cluster. Figure 9 shows typical fracture surfaces of internal crack initiation subjected to ultrasonic axial loading at *R* = −1 and 0.3, respectively. The inclusions were almost ellipsoidal or elongated in shape. From Figure 9, it can be seen clear ‘‘fish-eye’’ (FiE) patterns [37] for the whole fracture surfaces.

Based on the SEM/EDX analysis, the inclusions found at the fracture origins were MnS. This observation is different from the results reported by Bayraktar et al. [13], Li et al. [16], and Zettl et al. [38]. Although, MnS may be desired for improving machining properties of steels, they will also weaken the fatigue performance. In the present study, the MnS are almost elongated in shape. The influence of sulfide inclusion shape on crack initiation and propagation is discussed in the following sub-section.

### 3.4. Effect of Inclusions on the Fatigue Behaviors

**Figure 10:**
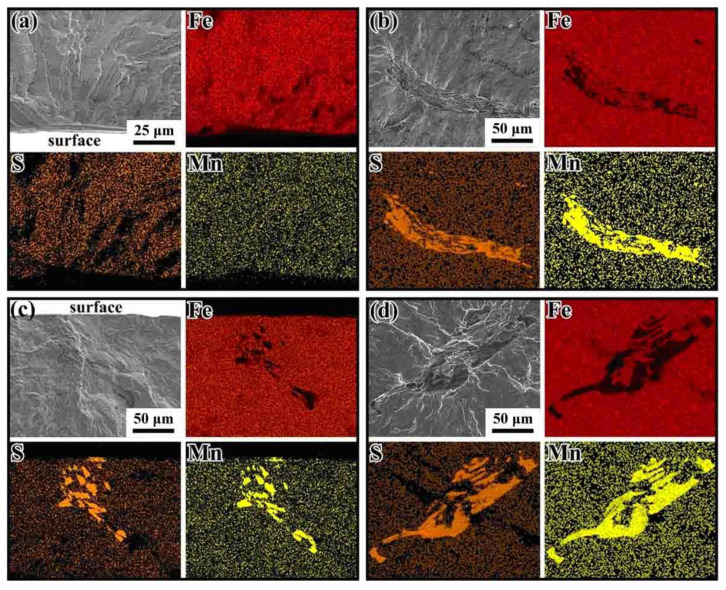
SEM images and EDX maps at the crack initiations on the fracture surface of failed specimens. (**a**) RB, surface slip; (**b**) UL, *R* = −1, internal elongated MnS; (**c**) UL, *R* = 0.3, surface elongated MnS; (**d**) UL, *R* = −1, internal elongated MnS.

Figure 10 shows SEM images and EDX mappings at the crack initiations on the fracture surface of failed specimens. As Figure 10b–d shows, the EDX measurements of inclusions in the fracture surfaces indicated that there were mainly elongated or ellipsoidal sulfides (MnS). The sulfide inclusions induced fatigue anisotropy on the fatigue crack initiation; the propagation has been confirmed in previous studies [39,40]. If the long axis of ellipsoidal sulfides is parallel to the cyclic loading direction, the projected area of MnS inclusions becomes small, and thus stress concentration produced by those sulfides is very little. If the long axis is perpendicular to the cyclic loading direction, the projection area of MnS becomes very large, and the higher stress will concentrate at the neighboring area of sulfides [23,24]; this will significantly degrade the fatigue strength. In our study, the orientation of MnS is perpendicular to the loading direction. However, as shown in Figure 9, the FiE area generated by crack initiation and propagation is almost round or elliptical in shape; this suggests that the crack actually originated from the sides of sulfide. In other words, the stress concentration may occur on the sides of the inclusion rather than the tip of sulfides.

**Figure 11:**
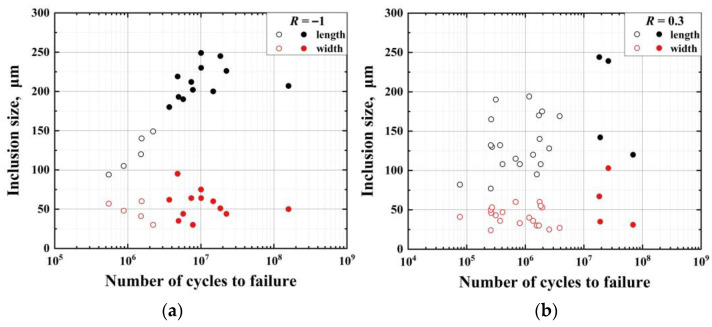
Relationship between inclusion size and fatigue life at (**a**) *R* = −1 and (**b**) *R* = 0.3. Hollow and solid symbols represent the locations of inclusion-inducing fatigue failure, hollow: surface; solid: interior.

In order to study the influence of MnS inclusions on crack initiation and propagation, some examples of finite element simulation was conducted to analyze the re-distributed stress due to the presence of the elongated inclusion. For this reason, Abaqus (6.13, 2013, DS Simulia, Providence, RI, USA) code was applied for this purpose. Firstly, we measured the size of the MnS inclusions at crack initiations on the fracture surface of failed specimens. Since the inclusions are all nearly elliptical, we measured the major axis (length) and minor axis (width) separately, and it is apparent that the equivalent diameter must lie between these two values. Figure 11 shows the size distribution in terms of length and width versus fatigue life *N*_f_. It can be seen that the lengths of MnS inclusions increase with the failure cycles, while the widths of MnS inclusions are less relevant to fatigue life. The lengths range from 100 to 250 μm, and the widths range from 25 to 75 μm. The ratio of length to width ranges from 2 to 6. The MnS inclusions are regarded as ellipsoidal models. They are all elongated spheres, in which the equatorial radius is the minor semi-axis length, and the polar radius is the major semi-axis length. Next, a cylinder model was established, which is with a diameter of 800 μm and a height of 300 μm simulating a part of the whole gauge length of the specimen. The cylinder and the ellipsoid are concentric and well bound with continuous stress and displacement.

**Figure 12:**
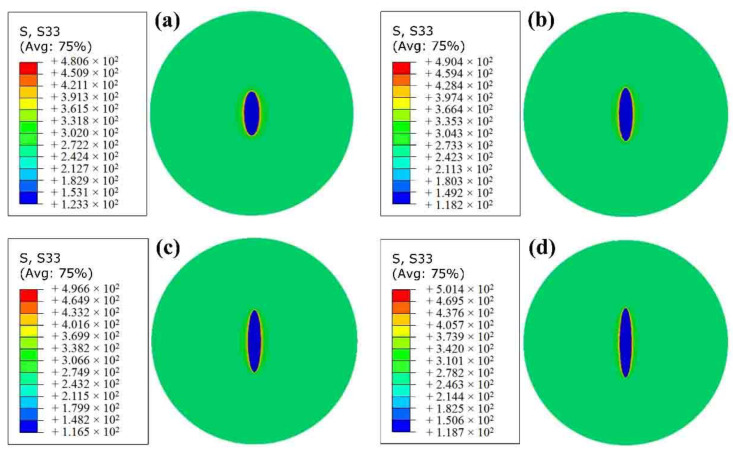
Stress distribution of the finite element models with different axial ratios (ARs) of elliptical MnS inclusion: (**a**) AR = 3, long axis of 170 μm; (**b**) AR = 4, long axis of 207 μm; (**c**) AR = 5, long axis of 240 μm; (**d**) AR = 6, long axis of 271 μm.

A constant value of 260 MPa of tensile stress was uniformly applied on top of the model. Four different axial ratios (AR) of major to minor ellipsoidal models were modeled. The set size of the ellipsoidal models was based on the detected size of the MnS inclusions in the specimens. Figure 12 shows the stress distribution of the finite element models with different axial ratios (namely 3, 4, 5, 6) of elliptical MnS inclusion with the same volume under the same load. The high stress concentrates on the sides of the elliptical MnS inclusion rather than the tip of the inclusion, led to crack initiation from the sides of the inclusions. Obviously, both the value and distribution of stress concentration will influence the behaviors of fatigue crack initiation and early growth in HCF and VHCF regimes. Thus, the elongated MnS inclusions may have a detrimental effect on the fatigue strength.

This effect explains the relationship of the MnS inclusion length and width versus fatigue life shown in Figure 11. It is known from the finite element simulations that the crack is prone to nucleate on both sides of the minor axis of the elliptical inclusion, so the minor axis length is the characteristic length of the after-formed crack. As shown in Figure 11, there is no apparent relationship between the length of the minor axis (i.e., the width of the elongated inclusion) and the number of failure cycles.

### 3.5. Microstructure Characterizations for the Internal Cases of Crack Initiation

**Figure 13:**
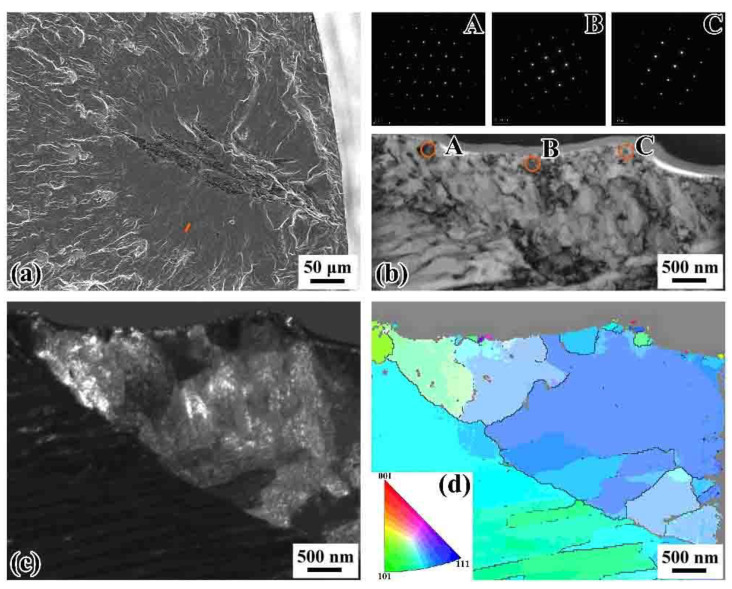
SEM and TEM (transmission electron microscopy) images of extracted longitudinal-section samples cut along the loading direction for a specimen that failed at the cycles of 1.01 × 10^7^ cycles under ultrasonic axial cycling at *R* = –1: (**a**) origin bar denoting FIB (focused ion beam) positioning location of TEM sample; (**b**) bright-field TEM image of the extracted cross-section sample, in which the labels A–C mark SAD (selected area electron diffraction) locations; (**c**) dark-field image of the longitudinal-section TEM sample; (**d**) the TKD (transmission Kikuchi diffraction) image.

Figure 13 shows the fracture morphology of SEM image and microstructure of TEM bright- and dark-field images of the extracted longitudinal-section sample from the FiE area of the failed specimen under ultrasonic loading at *R* = −1. The specimen failed at *σ*_a_ = 258 MPa, *N*_f_ = 1.01 × 10^7^ cycles, and *R* = −1. Figure 13b shows a detailed TEM morphology of the thin film, suggesting the original lamellar microstructure of pearlite. Moreover, the three SAD patterns at the selected circular domains composed of isolated spots indicate only one single grain within the SAD area. Note that the SAD patterns exhibit the microstructure just at local locations. The dark-field TEM image of Figure 13c shows the dispersed small bright humps along the fracture surface denoting the refined grains. It can be seen that the microstructure in the whole TEM sample was almost common lamellar pearlite. Figure 13d shows the TKD image of the sample. Some small grains were found on the fracture surface, indicating that grain refinement regions exist in the FiE area. The distribution of small grains is similar to the monotonic case of ductile fracture in some metallic materials [41].

**Figure 14:**
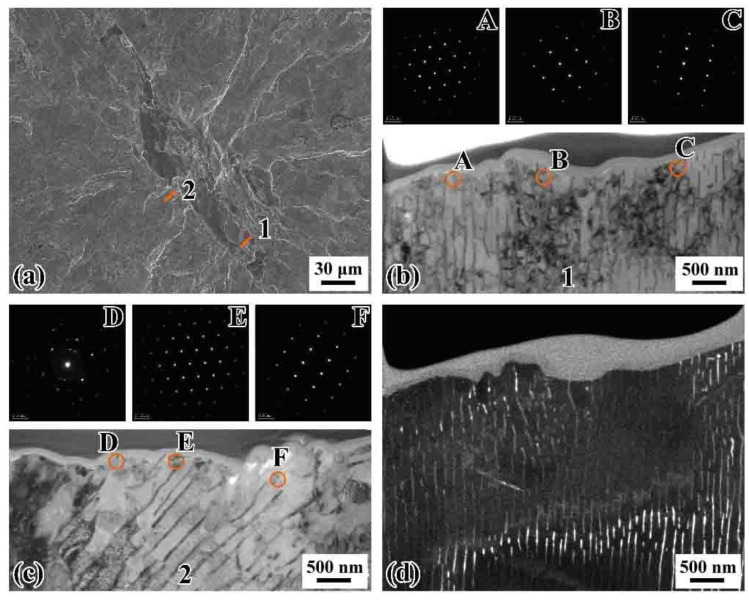
SEM and TEM images of extracted longitudinal-section samples cut along the loading direction for a specimen that failed at the cycles of 1.84 × 10^7^ cycles under ultrasonic axial loading at *R* = 0.3: (**a**) SEM image of the longitudinal-section positions; (**b**,**c**) bright-field images of TEM samples 1 and 2, respectively; the labels A–F mark the SAD locations; (**d**) dark-field image of TEM sample 1.

Figure 14 shows the fracture morphology of SEM and TEM images of two extracted longitudinal-section samples around the inclusion of the failed specimens under ultrasonic loading at *R* = 0.3. The specimen failed at *σ*_a_ = 202 MPa, *N*_f_ = 1.84 × 10^7^ cycles and *R* = 0.3. Figure 14b,c shows two bright-field morphology of lamellar pearlitic microstructure with SAD patterns exhibiting one set of isolated spots along the fracture surface. Figure 10d presents the dark-field image of TEM sample 1, indicating again the microstructure has the features of lamellar pearlite. These results suggest that no evident grain refinement presents in the FiE area at *R* = 0.3. Thus, we can infer that no microstructural changes occur underneath the fracture surface with crack initiation region for the specimens of VHCF failure at a positive stress ratio (*R* = 0.3).

## 4. Conclusions

In this paper, HCF and VHCF properties for a pearlitic wheel-steel were experimentally studied, and the conclusions are as follows:

1. All the S-N curves exhibit horizontal asymptotic shapes and have a clear fatigue limit. The fatigue limit (260–270 MPa) for *R* = −1 obtained by ultrasonic loading tests is almost 140–150 MPa lower than that obtained by rotating bending tests (400–410 MPa). The first reason is due to the temperature increase of the specimen at an ultrasonic frequency. The second reason is the larger control volume of ultrasonic fatigue specimens, which include a higher number of inclusions than rotating bending fatigue specimens. The fatigue limit at *R* = 0.3 is almost in the range of 195–205 MPa.
2. All fatigue cracks of the wheel-steel were originated from the surface microstructure for the rotating bending test, and no pre-existing inclusions were observed at the crack initiation sites. Whereas for ultrasonic loading tests, both surface and interior crack initiation occurred and all cracks initiated from MnS inclusions regardless of stress ratios, suggesting the sulfides as dominant factor for fatigue failure in HCF and VHCF regimes.
3. MnS inclusions at the crack initiation sites were examined by EDX. The finite element method was employed to study the influence of MnS inclusions’ shape on crack initiation and propagation. The results show that high stress concentrates on the sides of the elliptical MnS inclusion, rather than the tip of the inclusion, led to crack initiation from the sides of the inclusions.
4. TEM and TKD observations indicate that grain refinement regions exist in the FiE area of the VHCF failed specimens under ultrasonic loading at *R* = −1. However, no evident grain refinement is present in the FiE region at *R* = 0.3. This phenomenon suggests that refined grain appeared under the negative values of stress ratio and vanished under the positive values.

## Data Availability

Data available in a publicly accessible repository.
